# The Perspectives of Diabetes Educators and Dietitians on Diet and Lifestyle Management for Gestational Diabetes Mellitus: A Qualitative Study

**DOI:** 10.1155/2022/3542375

**Published:** 2022-06-22

**Authors:** Amber J. Hanks, Clare Hume, Siew Lim, Jessica A. Grieger

**Affiliations:** ^1^School of Public Health, University of Adelaide, Adelaide 5000, Australia; ^2^Adelaide Medical School, University of Adelaide, Adelaide, SA 5005, Australia; ^3^Monash Centre for Health Research and Implementation, Monash University, Clayton 3168, Australia; ^4^Robinson Research Institute, University of Adelaide, Adelaide, SA 5005, Australia

## Abstract

This study explores the knowledge and practice of diabetes educators and dietitians on diet and lifestyle management in women with gestational diabetes mellitus (GDM). Diabetes educators and dietitians were recruited from three maternity hospitals in Adelaide (Australia), through snowball and purposive sampling. Thirteen semistructured interviews were conducted, audio recorded, transcribed verbatim, and analysed for codes and themes. Four themes emerged: guidelines and resources, dietary intervention, management delivery, and communication. Diabetes educators and dietitians demonstrated consistent knowledge of nutritional management for GDM and uniform delivery methods. However, a lack of culturally diverse resources was highlighted, along with a lack of continuity of care across the multidisciplinary team. Barriers towards uptake of dietary intervention were reflected by diabetes educators and dietitians as women showing signs of guilt and stress and disengaging from the service. Further exploration on the knowledge and practice of diabetes educators and dietitians for GDM to best inform implementation strategies for knowledge translation of nutritional management is needed. The indication of language and cultural barriers and resources highlight an ongoing key priority area to support the care of women of ethnic minorities.

## 1. Introduction

Gestational diabetes mellitus (GDM) is the onset of diabetes or glucose intolerance during pregnancy. In Australia, the prevalence of GDM was at 15% in 2016–17 [1] which is intermediate to the reported prevalence worldwide of between 1% and 45% [2, 3]. Gestational diabetes mellitus is the most common metabolic disorder during pregnancy, contributing to a range of adverse maternal and infant health outcomes, both in the short term [4] and in the long term [5].

The current first-line clinical management of GDM is a healthy diet and exercise, based around the principles of optimal nutrition, weight control, physical activity, blood glucose monitoring, and insulin therapy. The conventional approach to diet therapy is carbohydrate restriction (30–40% of total energy intake), with the goal of blunting postprandial glucose [6, 7], to mitigate glucose-mediated fetal macrosomia. However, there are no universal guidelines for the management of GDM [8]. Instead, recommendations are provided by different health organisations, diabetes resources, and government sources [9–12]. While these recommendations typically follow the same principles regarding key health professionals to engage with for management of GDM and with the timing of blood glucose testing, there are inconsistent recommendations regarding the ideal blood glucose range, criteria to start medication, and frequency of GDM appointments, and there is limited information regarding dietary advice, follow-up appointments, and optimal weight gain.

There is a breadth of qualitative evidence exploring the experiences reported by women regarding their management of GDM, both within Australia and internationally. A systematic review of 10 studies highlighted that women with GDM found it difficult to change their eating habits because the recommended eating plan was so different from their previous dietary habits [13]. A systematic review of 41 studies revealed several barriers influencing a woman's ability to make lifestyle changes such as conflicting and confusing information given by health professionals, personal and professional commitments, women feeling like they do not have a choice in treatment, and the lack of understanding and knowledge [14]. Yet, while women report to face such challenges, there is far less information on healthcare providers who are key personnel that provide this information to women.

Among 12 general practitioners within Australia, many expressed discomfort about managing GDM on their own and reporting to lose touch with the patient for the remainder of the pregnancy [15]. Among 21 healthcare providers, there were reported challenges in providing care to South Asian women living in Australia, particularly regarding their self-management of blood glucose levels with lifestyle modification [16]. A small study of 6 diabetes educators which focused on their experiences of disadvantaged women with GDM highlighted that low socioeconomic status, low levels of education and literacy, and poor dietary habits significantly impacted on their understanding of GDM information, with a demonstrated need to target educational programs for women with low literacy [17]. Studies among health professionals internationally echo these findings, both in relation to women from resource-limited settings in South India [18] and in higher socioeconomic women in Singapore [19]. The experiences and perceptions of diabetes educators and dietitians, who are integral in providing education and promoting behaviour change, have not so far received a lot of attention. Further insight into the knowledge and practices, particularly regarding the diet and lifestyle management of women with GDM, is essential to the development of meaningful care programs. This will support improved and consistent care, increase the usefulness of information given to women with GDM, and optimise the overall health of pregnant women and their children.

This study is aimed at exploring the knowledge and practice of diabetes educators and dietitians on diet and lifestyle management in women with GDM and, specifically, the barriers and facilitators influencing practice.

## 2. Subjects, Materials, and Methods

### 2.1. Design

This study, using a qualitative design, investigated the “how” and “why” about beliefs, professional procedures, facilitators, and barriers influencing the diet and lifestyle interventions given to women with GDM. Diabetes educators and dietitians were recruited to take part, either face-to-face or by video conference, in semistructured interviews.

### 2.2. Sampling and Recruitment

Diabetes educators and dietitians were recruited using purposive and snowballing sampling [20] within three public maternity hospitals in Adelaide (South Australia, Australia). In total, 22 individuals were contacted to participate. Fourteen (64%) potential participants were directly contacted by email, and eight (36%) were obtained by a snowball effect. Nine diabetes educators and dietitians did not respond when directly contacted. Correspondence with hospital reception also occurred to obtain potential participants, but no participant was recruited through this method. Inclusion criteria were diabetes educator or dietitian currently managing GDM patients in a maternity hospital in Adelaide. As participants were purposefully selected, no participants were excluded. Each diabetes educator or dietitian was given information on the study and provided informed consent (signed or audiotaped) before each interview. Participants were recruited until data saturation was reached, which was at 13 interviews, and interviews were conducted between June and September 2021.

### 2.3. Data Collection

This study was guided by the consolidated criteria for reporting qualitative research guidelines [21], to enhance research validity (purposeful sampling, audit trail), rigour (data saturation, ethics approval), credibility (member checking), and generality (inclusion criteria). The interview originally utilised a reflexive approach [22], allowing flexibility for the researcher to either elaborate or clarify certain responses without a preestablished assumption. The interview approach ensured that questions were pertinent, open ended, and exploratory in nature. Basic demographic data including gender, age, years and place of practice, and frequency of GDM encounters per week were collected with a questionnaire before the interview started. The interviews explored the current perceptions, knowledge, and procedures for lifestyle management for women with GDM. Each diabetes educator and dietitian described how often they manage a woman with GDM, how and what advice and information are given at each appointment, facilitators and barriers in managing GDM during and outside of appointments, and any recommendations to address these issues. The semistructured interviews were conducted by one researcher (AH), following training by (JAG) and a PhD student experienced in qualitative research (JM). A single interviewer allowed for continual and simultaneous data collection, crosschecking of themes, and confirmation of data saturation. Interviews were audio recorded and conducted in locations convenient to diabetes educators and dietitians such as consulting rooms, libraries, or by video conference.

### 2.4. Data Analysis

Deidentified audio recordings were transcribed verbatim, and transcriptions were reviewed by AH for accuracy. Transcripts were thematically analysed and coded using NVivo version 12 Plus (Windows) 2018 QSR International Pty. Ltd. Software. Using the Braun and Clarke method [23], familiarising the transcript is the first step in data analysis, followed by the identification of elements of interest in the data. These initial codes were then linked to create themes. Final themes were reviewed to ensure that they were reflective of the original transcript and of the research question. AH coded all transcripts, and JAG independently coded a third of the transcripts, which were then reviewed and compared with coding by AH to ensure consistency and reliability. Discussions between investigators were conducted three times to gain a consensus on codes and themes.

### 2.5. Ethical Approval

Ethical approval was granted by the women's and children's ethics committee, with site-specific approval being obtained from each participating hospital. The ethics approval number is 2021HRE/00128 (date of approval: 25/04/2021).

## 3. Results

Seven diabetes educators and six dietitians participated from across three public maternity hospitals in Adelaide. Interviews ranged in the duration from 18 to 54 minutes. Participants were aged between 24 and 53 years, with 0.5–25 years of experience (Table 1). At least 70% of diabetes educators and dietitians reported to see patients in a group setting. Dietitians reported following up with 26% of patients after the initial appointment, whereas diabetes educators had weekly follow-up appointments with each patient unless they declined.

Interviews revealed four themes: guidelines and resources, dietary intervention, management delivery, and communication (Table 2).

### 3.1. Theme 1: Guidelines and Resources

Diabetes educators and dietitians demonstrated an awareness of GDM guidelines within Australia, including from The Australasian Diabetes in Pregnancy Society and the National Diabetes Service Scheme which were common resources used across all hospitals.

“*We use NDSS [National Diabetes Services Scheme] a lot for our general information. And ADIPS would be our biggest resource*” (F-dietitian—52 yrs).

There was clear acknowledgement of recommendations for carbohydrate intake, with information delivered either as grams of carbohydrates per meal, per snack, or total carbohydrate intake. However, there were differences in the use of additional resources and how they were developed.

“*I think it's the diabetes nurse, or it could be the Allied health assistance who puts together the packs*” (F-diabetes educator—37 yrs).

“*Those resources, they are developed by us [hospital], so they are our own resources that we created over the years*” (F-dietitian—33 yrs).

“…*some of them are more just nutrition in pregnancy rather than specifically for gestational diabetes*” (M-diabetes educator—53 yrs).

Furthermore, it was synonymous that there was a lack of cultural resources specific to culturally and linguistically diverse women, but also dietitians who could educate them.

“*More cultural specific dietitians…. that are diet specific to that culture*” (M-diabetes educator—53 yrs).

“*Resources for non-English speaking, for Afghan…. Nepalese would be a good one as well*” (F-dietitian—33 yrs).

“*We've only got a narrow selection of interpreted resources… we've got that interpreted in three languages*” (F-dietitian—32 yrs).

“…*when I get a non-English speaking going my clinic, I look for the [notes indicating her] language and quite often there isn't that particular language on there*” (F-diabetes educator—37 yrs).

### 3.2. Theme 2: Dietary Intervention

#### 3.2.1. Culturally Suitable Advice

Participants highlighted the importance of keeping women on a culturally appropriate diet and working with what the women are currently eating. Dietitians often stated that they did not change the type of food that women were eating, instead focusing on reducing women's carbohydrate intake.

“*A good dietitian uses foods that they like and doesn't try to change their whole diet, because then they'll say this is too hard*…” (F-dietitian—24 yrs).

“*Having an understanding of what cultural foods they might be having and then what carbohydrate content is in those foods… Indian background women will be having like roti, chapati and that kind of stuff*…” (F-dietitian—28 yrs).

While participants would try to be sensitive towards cultural diversity, the social and family contexts proved challenging.

“…*cultural background is very difficult…I'm from an Italian background and if you say no to eating a big plate of pasta you're offending*…” (F-dietitian—33 yrs).

“*Cultural foods are very different to what our dietitians are used to…[they] have more difficulties making those adjustments*” (M-diabetes educator—53 yrs).

#### 3.2.2. Restrictive Eating

Participants stated that dietary advice was the first management intervention given to women diagnosed with GDM, with medical intervention occurring only if blood glucose levels remained high. Nevertheless, the dietary advice was also speculated to make women feel anxious and stressed with some fear of starting insulin.

“*They feel a lot of stress,… to try and get these perfect sugar levels, they can put a lot of stress on themselves and feel a lot of guilt if they're not getting the numbers that they want*” (F-diabetes educator—30 yrs).

Diabetes educators and dietitians also speculated that the woman's anxiety and stress to get blood glucose levels in a healthy range may result in them restricting their diet, particularly their carbohydrate intake.

“*Sometimes, I think some women might be quite carb restrictive and so that's why their sugar levels are appearing to all to be in normal range and babies measuring really quite small*” (F-diabetes educator—30 yrs).

“…*we do find they cut out all carbohydrates completely and then it's really about re-educating and reinforcing*” (F-dietitian—28 yrs).

#### 3.2.3. Insulin

Medication, generally either insulin or metformin, was typically suggested to women after dietary changes were recommended and implemented and if they were still having consistently high blood glucose levels. It was indicated that some women, no matter what dietary changes occurred, would require medication.

“…*Depends on the women, if they are doing all of the recommended recommendation with diet and lifestyle… so that's when we have to go on medication*” (M-diabetes educator—53 yrs).

“*And I'd say roughly 50% would end up needing insulin education*” (F-diabetes educator—30 yrs).

However, the apprehension towards starting insulin was one of the biggest barriers in their appointment sessions and was suggested to contribute to women not engaging with the service.

“*You'll notice that they need to be on insulin, but they haven't been contacting the diabetes educators to report their levels*” (F-dietitian—28 yrs).

“*Some women expressed to me that they don't want to go on medication as well in the fear of medication*” (F-dietitian—27 yrs).

However, it was also reported that once women started insulin, they often felt relieved and had a positive experience. If a woman ended up in the service for a second time, they were generally less hesitant to go on insulin again.

“*They can tend to find starting insulin a relieving experience because they start eating more and feeling more nourished…they see numbers that they want*…” (F-diabetes educator—30 yrs).

“…*if they've had insulin and things before that… I think they're just more aware…and less anxious*” (F-diabetes educator—39 yrs).

### 3.3. Theme 3: Management Delivery

#### 3.3.1. Adequacy of Appointment Sessions

Diabetes educators and dietitians indicated that the current time allocated for group appointments and one-on-one appointments (30–60 minutes) was adequate to get through majority of the required information and answer questions, whilst assuming that it was not overbearing for the women.

“*It's not so much timing, we've got plenty of time to go through that with them*” (F-diabetes educator—29 yrs).

“*I would say at least 45 minutes up to an hour if there's lots of questions*” (F-dietitian—32 yrs).

Additionally, many diabetes educators, whilst being allocated shorter phone appointment sessions or email conversations, believed that the level of service that they provide is adequate due to the frequency of them communicating with women.

“*I feel so because they get at least a minimum of weekly phone contact where we're able to actually follow up questions and ask*…” (F-diabetes educator—30 yrs).

However, the wait time to commence the service, particularly with dietitians, was indicated to be lengthy and was determined mainly by women's risk factors and referral indicators. Diabetes educators from 2 of the 3 hospitals stated that women would remain in their service until birth, generally around 12 weeks of pregnancy, whilst one stated that they would be discharged from the service once their glucose levels were in an adequate range.

“…*we see them in three to four weeks, the high BMIs. If they're in early diagnosis, we try and see them within about six weeks*” (F-dietitian—28 yrs).

“*We don't even have spots. We're meant to see these ladies within a week that are high risk and often we can't even get them in within a week*” (F-diabetes educator—37 yrs).

Additionally, follow-up appointments with a dietitian are offered if a dietitian believed a woman needed further support or information. Weekly contact, either face-to-face, email, or phone, with a diabetes educator was also encouraged. It was recognised that while there was consistency in the management of GDM during pregnancy across the three hospitals, follow-up appointments were inadequate, with a different mode of delivery for non-English speaking women.

“*No, it doesn't meet the Australian guidelines for what we should be doing in terms of follow up by any means, we are nowhere near it. I don't think it meets the needs*” (F-dietitian—27 yrs).

“*Not everyone gets a follow-up, so it's only if we feel that they need it*” (F-dietitian—33 yrs).

“*We have two pathways, if English speaking it is the group. If you're non-English speaking it is one-to-one*” (F-dietitian—52 yrs).

#### 3.3.2. Delivery Methods

All diabetes educators and dietitians indicated that they conveyed their information to women, either in a group or in a one-on-one setting using a visual and interactive approach. This was conducted with the use of PowerPoint, booklet, food models, quizzes, writing activities, and/or questions.

“*We just speak and try and make it as interactive as we can…we try and not just have it as a PowerPoint*” (F-dietitian—28 yrs).

“*We have activities throughout…a number of quizzes particularly covering blood sugar levels and what we are aiming for… label reading*…” (F-dietitian—27 yrs).

However, whilst the interactive sections of the sessions were deemed to be inviting, it was commonly mentioned that the information-dense sections had the opposite effect.

“…*I'll only get up to half, a quarter of the way through the education and I'll see…that they're glazing over, they're … just completely disengaged I'll say*” (F-dietitian—28 yrs).

#### 3.3.3. Individualised Lifestyle Counselling

There was a common consensus across diabetes educators and dietitians about the benefit of individualising and approaching appointments in an adaptive manner. Those who worked with patients in individual appointments reported having to monitor and change their appointment sessions depending on the requirements of their patient such as literacy level, social and cultural circumstances, and a woman's motivation, and the time required for individualising sessions was a barrier.

“*I sort of asked them how best do you learn? How can I accommodate what you need from this session*” (F-diabetes educator—37 yrs).

“*It's just a more personalized approach, so you can go through their specific diet…you can count their carbohydrates….and personalize the plan*” (F-dietitian—32 yrs).

“*We try to be as, I guess as holistic as possible, but sometimes need to rein it in to just the gestational diabetes issues*” (F-dietitian—32 yrs).

“*You just don't have the time and it's really sad that you feel rushed … you've gotta keep reminding yourself that this is a patient who has a problem*” (F-diabetes educator—37 yrs).

### 3.4. Theme 4: Communication

#### 3.4.1. Teamwork

Many diabetes educators and dietitians, mainly those conducting one-on-one appointments, praised the GDM network within their hospital and strongly emphasised the importance and need for having a multidisciplinary approach. They rationalised the importance of a team environment due to the increased access to resources and expertise and reinforcement of key information.

“…*we can email the obstetric medicine staff if we need to. We have our endocrine registrar who also helps us. And some of the key midwives in the clinic… the dietitian, if there's a question or someone wants to be referred for an individual session*” (F-diabetes educator—50 yrs).

“*We are very much multidisciplinary team here…we work with the diabetes nurse educators…midwives…the diabetes educators and dietitians work pretty alongside each other*” (F-dietitian—33 yrs).

On the contrary, poor communication between health professionals was a clear barrier affecting continuity of care. There was reported difficulty when women saw a different healthcare professional who would not seem to understand about GDM and would suggest different blood glucose level targets.

“…*you've got so many people who are involved and so people are always giving advice mostly from their professional perspective… you miss things that are going on*” (F-diabetes educator—30 yrs).

“*Probably the obstetric team, because they will just do the OGTT and refer to us, often the patients that get referred to us either haven't been told they've got gestational diabetes, or they've got no understanding of what happens next*” (F-dietitian—52 yrs).

“*The doctors lack of awareness on what is an appropriate BGL target for someone who is pregnant with GDM, not someone who is type 2 and not pregnant*” (M-diabetes educator—53 yrs).

#### 3.4.2. Communication with Patients

Education and empowering women to make their own choices were commonly suggested to be one of the most influential and helpful aspects in managing a woman's GDM. Participants identified effective communication and education as a tool to increase motivation and encourage women to participate with the service.

“*Informing them and then feeling like [they are] empowered to self manage*” (F-diabetes educator—39 yrs).

Diabetes educators and dietitians also identified that having a personal conversation and a flexible approach regarding appointment discussions was important in making the women feel valued and listened to. However, the high frequency of daily appointments with strict time allowances sometimes limited their ability to adapt and change appointments to suit the personal requirements of their patients.

“*Sometimes it's just explaining why we're here*” (F-diabetes educator—30 yrs).

“*There's just been times where they've come to an appointment, but they're just not in the place where they can take on any real information. So, in those times I might just sit with them and chat about things generally to see how they're going*” (F-dietitian—28 yrs).

Effective communication was not always possible among non-English-speaking women. Interpreters are often used in one-on-one appointments to allow diabetes educators or dietitians to communicate with their patients, though interpreters did not necessarily translate the appointment adequately or were not fully engaged throughout the session.

“*We go through two companies for interpreting but sometimes we have interpreters where their English is so poor that I don't even know if they are interpreting what I am saying to a patient*” (F-dietitian—52 yrs).

“*The interpreters might be on their phone, or not interpreting properly”* (F-dietitian—52 yrs).

## 4. Discussion

The current study contributes to the limited literature that has explored the experiences and perceptions of diabetes educators and dietitians regarding diet and lifestyle management for women with GDM. The current exploratory research was timely given the increasing prevalence of GDM and due to the growing strain and challenges placed on the healthcare sector [1, 24]. Four interconnected themes were found: guidelines and resources; dietary intervention; management delivery; and communication.

The dietitians and diabetes educators that we studied were familiar with appropriate guidelines to manage GDM, and there was consistency across participants for carbohydrate recommendations. This is important because many guidelines recommend health education sessions and a dietitian to give nutrition therapy [25]; they are often the first health professional whom women are referred to following a diagnosis of GDM [15] and are commonly seen as the most important facilitator to improve successful adaptations to dietary recommendations [26]. Furthermore, it was encouraging to have diabetes educators and dietitians report consistent methods for the delivery of nutritional information and their attitudes towards the need for individualised education, as per the current antenatal model within Australia [27]. However, there was a common indication about the lack of resources for culturally and linguistically diverse women.

The self-reported knowledge of the current guidelines among our diabetes educators and dietitians provides a novel aspect to the many other studies among healthcare professionals who report challenges towards the nutritional management of GDM. Only one other qualitative study from Australia was found, which, among healthcare practitioners (that included dietitians and diabetes educators) reported to struggle to provide information on how to successfully self-manage GDM, with the diversity of their cliental (South Asian women) seen as a major challenge [16]. Among health professionals in New Zealand, despite their understanding of the importance of nutrition for GDM, there were challenges around the nutrition guidelines for women with a lower body mass index [28] and regarding minimum carbohydrate intake [29]. Furthermore, in low-resource settings, there are limited healthcare professionals who uniformly adhere to national recommendations of the management of GDM, mainly due to the lack of a qualified workforce [18], with diabetes educators looking for ways to make the guidelines accessible and meaningful [17]. The lack of cultural-specific resources and the limited access to interpreters described in our study likely impact the uptake of knowledge among women from ethnic minorities. There is sufficient evidence in women who had GDM highlighting information provision to be unsatisfactory and limiting [16, 28, 30], with women having limited ability, self-efficacy, and understanding to adopt nutrition recommendations and manage nutrition choices [31, 32]. To improve knowledge translation and uptake, supporting awareness of cultural resources and enhancing dissemination strategies for GDM educational material are needed for healthcare providers and patients.

While facilitators towards nutrition guidelines were evident, our study highlights a clear disconnect between knowledge and translation. A recent analysis of 15 members of the Australasian Diabetes in Pregnancy Society Australia and New Zealand described different antenatal models of care with a range of local innovations for women with GDM [27]. However, no state-wide or national strategy has been developed to manage this. It is too simplistic to conclude that culturally relevant resources will increase women's understanding and ability to engage with GDM management plans. We need further exploration of how healthcare professionals, and specifically diabetes educators and dietitians, envisage that there could be improved uptake of knowledge for all women and what resources they require to do this. Training specific to management of GDM would also ensure consistency across healthcare professionals for diabetes diagnoses.

Barriers towards uptake of dietary intervention were revealed by diabetes educators and dietitians with women tending to follow nutritional advice out of fear of the risks for their baby, fear of insulin/needles and disengaging from the service, and with women often showing signs of guilt and stress if they did not meet the recommended glucose targets. Layered on this was the notion that restrictive eating was a concern, particularly with carbohydrate intake. Concerns of fear, anxiety, and restrictive eating are also reported by women themselves following a diagnosis of GDM [14, 30, 33], but they also have identified coping strategies including sticking to a simple diet [34], social support from families, and expert advice and psychological support from healthcare providers [35] and free monitoring and access to education about glucose monitoring [36]. Evidence among health professionals has demonstrated training packages increase women's self-efficacy [37] and the potential inclusion for mobile health technology, so that women can access repeated information at their own convenience [38]. Other studies have revealed that providing empathy [39], emotional support, and understanding different ways of communicating [40] may create opportunities to share perspectives and overcome barriers of fear and anxiety. Effective communication and education were facilitators to increase motivation and encourage women to participate with the current dietetic service. There is an ongoing need to acknowledge psychosocial challenges, to create meaningful and supportive interventions, to facilitate patient engagement, and to improve GDM management in women.

A range of teamwork communication barriers was identified. Consistent with previous studies among healthcare professionals [15, 27, 41–43], reported barriers included waiting time for appointments, the lack of continuity of care, and the discrepancy between who should and who does receive any follow-up care throughout the pregnancy. While dietitians and diabetes educators were reported to work well together and they were amongst a “multidisciplinary team,” there was a lack of effective communication for their particular role. Survey data from Australia also shows disparity in management principles, including blood glucose targets, initial management strategies, and role perceptions [44]. Sadly, this is not restricted to the management of GDM; insufficient use of allied health and inadequate information provision are common to other women's conditions including polycystic ovary syndrome [45], but also type 2 diabetes [46], and in obesity and weight management [47, 48]. We have consistent evidence, but we now need solutions. By understanding the current practices, health services can begin to develop new models of care that can improve efficiencies, costs, and patient care that is streamlined across all regions and health services in Australia.

### 4.1. Recommendations for Future Practice

This study has generated a foundation for further exploration on the current management practices for GDM given by diabetes educators and dietitians. While evidence is growing, particularly for the use of mobile health technology, further work is needed on how barriers for knowledge translation can be broken down, but also the development of feasible strategies to support crossdisciplinary communication and continuity of care. Study outcomes emphasise the ongoing need for culturally effective resources to be made available for health professionals, with strategies to promote uptake for women with GDM. Increased availability to dietitians and access to follow-up appointments are also recommended to build healthier attitudes towards nutritional management, uptake of diet and lifestyle advice, and potentially the reduced need for medical intervention. The psychosocial concerns of anxiety and fear implicate the potential role that psychologists may play regarding the nutritional management in women with GDM.

### 4.2. Strengths and Limitations

This study provides important information on knowledge and experiences for GDM management among diabetes educators and dietitians in Adelaide (Australia). Results provide novel aspects regarding the consistency in knowledge and nutritional management and builds on the ongoing communication barriers and lack of culturally diverse resources that are reported by health professionals. The current study was conducted across all public maternity hospitals in Adelaide, which allowed for different sociodemographic areas to be included. However, it did not include private hospitals and medical clinics; thus, results may not be generalisable to all of South Australia. The sample included a young participant group (median 31 years) who had limited experience in their role (69%, <5 yrs). This contrasts to the reported characteristics of a 2007 Australian Diabetes Educators Association survey (*n* = 212) on attributes and barriers to effective teaching and learning, of whom 78% were aged 40 years or over and whom 72% had ≥4 years in diabetes education [49]. However, despite these differences, our results shed new light on facilitators and barriers to nutritional management among diabetes educators and dietitians and highlights some similar themes between our study and others. Perspectives of women who have had GDM would additionally provide a balanced view on the current GDM diet and lifestyle management.

## 5. Conclusion

This study generated four key themes involving guidelines and resources, dietary intervention, management delivery, and communication. It is evident that diabetes educators and dietitians were well trained with consistent delivery methods but there was a large gap in translation and continuity of care. Our study highlights the need for further exploration on the knowledge and practice of diabetes educators and dietitians for GDM to best inform implementation strategies for knowledge translation of nutritional management. The indication of language and cultural barriers and resources highlight an ongoing key priority area to support the care of women of ethnic minorities.

## Figures and Tables

**Table 1:** Descriptive characteristics among the interviewed diabetes educators and dietitians.

Characteristic	*n* (%)
Age (years), median (range)	31 (24–53)
Female/male	12 (92%)/1 (8%)
Dietitians	6 (46%)
Diabetes educators	7 (54%)
Years worked, median (range)	4.5 (6 months–25 years)
<5 years	9 (69%)
6–15 years	2 (15%)
>16 years	2 (15%)
Diabetes educators	
New patients, weekly, median (range)	Group education: 20 (5–25), individual clinic: 3.5 (2–6)
Follow-ups, weekly, median (range)	46 (15–100)
Dietitians	
New patients, weekly, median (range)	Group education: 13.5 (5–24), individual clinic: 5.5 (4–6)
Follow-ups, weekly, median (range)	5 (0–12)

**Table 2:** Barriers and facilitators to current management practices of diabetes educators and dietitians.

Subtheme	Facilitator/barrier	Quotes
Theme 1: guidelines and resources		
—	Facilitator	“We use NDSS (National Diabetes Services Scheme) a lot for our general information. And ADIPS would be our biggest resource” (F-dietitian—52 yrs). “Australian guidelines for gestational diabetes and counting carbohydrates, so around 30-45 grams of carbohydrates per main meal, 15-30 grams carbohydrates for a snack” (M-diabetes educator—53 yrs).
	Barrier	“Those resources, they are developed by us [hospital], so they are our own resources that we created over the years….some of them are more just nutrition in pregnancy rather than specifically for gestational diabetes” (M-diabetes educator—53 yrs). “I think it's the diabetes nurse, or it could be the allied health assistance who puts together the packs” (F-diabetes educator—37 yrs). “We've only got a narrow selection of interpreted resources….we have got that interpreted in three languages…in Chinese, Vietnamese and Arabic” (F-dietitian—32 yrs). “…when I get a non-English speaking going my clinic, I look for the…[notes indicating her] language and quite often there is not that particular language on there” (F-diabetes educator—37 yrs).
Theme 2: dietary intervention		
Culturally suitable advice	Facilitator	“I guess having an understanding of what cultural foods they might be having and then what carbohydrate content is in those foods…Indian background women will be having roti, chapati and that kind of stuff…” (F-dietitian—28 yrs). “A good dietitian uses foods that they like and does not try to change their whole diet because then they'll say this is too hard…” (F-dietitian—24 yrs).
	Barrier	“…cultural background is very difficult, I'm from an Italian background and if your say no to eating a big plate of pasta you are offending” (F-dietitian—33 yrs). “Cultural foods are very different to what our dieticians are used to…[they] have more difficulties making those adjustments” (M-diabetes educator—53 yrs).

Restrictive eating	Barrier	“They feel a lot…of stress, and…need to…try and get these perfect sugar levels, they can put a lot of stress on themselves and feel a lot of guilty if they are not getting the numbers that they want” (F-diabetes educator—30 yrs). “I think some women might be quite carb restrictive and so that's why their sugar levels are appearing to all to be in normal range and babies measuring really quite small” (F-diabetes educator—30 yrs). “…we do find that cut out all carbohydrates completely and then it's really about re-educating and reinforcing” (F-dietitian—28 yrs).

Insulin	Facilitator	“They can tend to find starting insulin a relieving experience because they start eating more and feeling more nourished…they see numbers that they want…” (F-diabetes educator—30 yrs). “…if they have had insulin and things before that…I think they are just more aware of things and less anxious” (F-diabetes educator—39 yrs).
	Barrier	“…depends on the women, if they are doing all of the recommended recommendation with diet and lifestyle… that's when we have to go on medication” (M-diabetes educator—53 yrs). “Some women expressed to me that they do not want to go on medication as well in the fear of medication” (F-dietitian—27 yrs). “You'll notice that they need to be on insulin, but they have not been contacting the diabetes educators to report their levels” (F-dietitian—28 yrs).
Theme 3: management delivery		
Adequacy of appointment sessions	Facilitator	“It's not so much timing, we have got plenty of time to go through that with them” (F-diabetes educator—29 yrs). “I would say at least 45 minutes up to an hour if there's lots of questions” (F-dietitian—32 yrs). “I feel so because they get at least a minimum of weekly phone contact where we are able to actually follow up questions and ask so…they are getting quite regular contact with access to diabetes education” (F-diabetes educator—30 yrs).
	Barrier	“…we see them in three to four weeks, the high BMIs. If they are in early diagnosis, we try and see them within about six weeks…” (F-dietitian—28 yrs). “We do not even have spots. We're meant to see these ladies within a week that are high risk and often we cannot even get them in within a week…” (F-diabetes educator—37 yrs). “Not every English-speaking girl gets offered a review, the review is only after their sugars are high” (F-dietitian—32 yrs). “No, it does not meet the Australian guidelines for what we should be doing in terms of follow up by any means, we are nowhere near it. And I do not think it meets the needs” (F-dietitian—27 yrs). “We do not have any set routine for how much. Not everyone gets a follow-up, so it's only if we feel that they need it” (F-dietitian—33 yrs). “We have two pathways, if English speaking it is the group. If you are non-English speaking it is one-to-one” (F-dietitian—52 yrs).

Delivery mode and resources	Facilitator	“We just speak and try and make it as interactive as we can…we try and not just have it as a PowerPoint and try and make it interactive” (F-dietitian—28 yrs). “We have activities throughout as well, so a number of quizzes particularly covering blood sugar levels and what we are aiming for…we also have an activity about label reading” (F-dietitian—27 yrs).
	Barrier	“…I'll only get up to half, a quarter of the way through the education and I'll see…that they are glazing over they are …just completely disengaged I'll say” (F-dietitian—28 yrs).

Individualised lifestyle counselling	Facilitator	“I sort of asked them how best do you learn? How can I accommodate what you need from this session” (F-diabetes educator—37 yrs). “It's just a more personalized approach, so you can go through their specific diet…you can count their carbohydrates….and personalize the plan” (F-dietitian—32 yrs).
	Barrier	“We try to be as, I guess as holistic as possible, but sometimes need to rein it in to just the gestational diabetes issues” (F-dietitian—32 yrs). “You just do not have the time and it's really sad that you feel rushed…you have gotta keep reminding yourself that this is a patient who has a problem” (F-diabetes educator—37 yrs).
Theme 4: communication		
Teamwork	Facilitator	“…We can email the obstetric medicine staff if we need to. We have our endocrine registrar who also helps us. And some of the key midwives in the clinic…the dietitian, if there's a question or someone wants to be referred for an individual session” (F-diabetes educator—50 yrs). “We are very much multidisciplinary team here…we work with the diabetes nurse educators…midwives…the diabetes educators and dietitians work pretty alongside each other” (F-dietitian—33 yrs).
	Barrier	“I mean, I suppose sometimes you have got so many people who are involved and so people are always giving advice mostly from their professional perspective. But you are not getting that whole collaborative all in one go kind of…you miss things that are going on” (F-diabetes educator—30 yrs). “Probably the obstetric team, because they will just do the OGTT and refer to us, often the patients that get referred to us either have not been told they have got gestational diabetes, or they have got no understanding of what happens next” (F-dietitian—52 yrs). “The doctors lack of awareness on what is an appropriate BGL target for someone who is pregnant with GDM, not someone who is type 2 and not pregnant” (F-dietitian-32 yrs).

Communication with patients	Facilitator	“Informing them and then feeling like [they are] empowered to self manage” (F-diabetes educator—39 yrs). “Sometimes it's just explaining why we are here.…” (F-diabetes educator—30 yrs).
	Barrier	“There's just been times where they have come to an appointment, but they are just not in the place where they can take on any real information. So, in those times I might just sit with them and chat about things generally to see how they are going” (F-dietitian—28 yrs). “We go through two companies for interpreting but sometimes we have interpreters where their English is so poor that I do not even know if they are interpreting what I am saying to a patient” (F-dietitian—52 yrs). “The interpreters might be on their phone, or not interpreting properly” (F-dietitian—52 yrs).

## Data Availability

Deidentified data transcripts are available upon request to the corresponding author.

## Abbreviations

GDM: Gestational diabetes mellitus
BMI: Body mass index
OGTT: Oral glucose tolerance test
BGL: Blood glucose level
ADIPS: Australasian Diabetes in Pregnancy Society.
